# Prevalence and antibiotic resistance profile of UTI-causing uropathogenic bacteria in diabetics and non-diabetics at the Maternity and Children Hospital in Jeddah, Saudi Arabia

**DOI:** 10.3389/fmicb.2024.1507505

**Published:** 2024-11-28

**Authors:** Peter F. Farag, Hamzah O. Albulushi, Mohammed H. Eskembaji, Mohammad F. Habash, Mohammed S. Malki, Muayad S. Albadrani, Ahmed M. Hanafy

**Affiliations:** ^1^Department of Microbiology, Faculty of Science, Ain Shams University, Cairo, Egypt; ^2^Laboratory Department, Medical Center, Taibah University, Al-Madinah, Saudi Arabia; ^3^Department of Oncology and Medical Histology Pathology, Medical Sciences College Taibah University, Al-Madinah, Saudi Arabia; ^4^Microbiology Laboratory, Maternity and Children Hospital, Jeddah, Saudi Arabia; ^5^Makkah Health Cluster, Ministry of Health, Makkah, Saudi Arabi; ^6^Department of Family and Community Medicine and Medical Education, College of Medicine Taibah University, Al-Madinah, Saudi Arabia

**Keywords:** urinary tract infection, female patients, etiological profiles, antibiotic resistance, age categories

## Abstract

**Introduction:**

One of the most prevalent and recurrent infectious diseases that can range from moderate to fatal is urinary tract infection (UTI). Broad-spectrum antibiotics are the only management strategy for UTIs in ambulators and hospital stays. Due to the ongoing emergence of antibiotic resistance among uropathogens, there is a need for proper selection of antibiotics for empirical therapy against UTIs. This study aimed to compare the etiological profiles and antibiotic susceptibility patterns between diabetic and non-diabetic UTI female patients from the Maternity and Children Hospital in Jeddah, Saudi Arabia.

**Methods:**

Urine samples from different age categories of female UTI patients were collected from January 2021 to June 2023. The positive urine cultures with a single pathogen were selected and all bacterial isolates were identified by MALDI-TOF/MS system. Antibiotic susceptibility testing was done using VITEK-2. Our study included 2,245 female patients, of which 1825 (81%) were non-diabetic and 420 (19%) were diabetic.

**Results:**

The results showed a significant relationship (*p* = 0.00063) between the average age and the number of diabetic UTI patients. Gram-negative bacilli were more dominant (84.7%, n = 1903) than gram-positive cocci (15.3%, n = 342). *Escherichia coli* (40.8%) was the most prevalent pathogen identified with a significant (*p* < 0.0001) increase in non-diabetic (45.26%) than diabetic UTI patients (21.43%). *Proteus mirabilis* (10.2%) and *Pseudomonas aeruginosa* (8.7%) followed *E. coli* in pathogen distribution. Among gram-positive species, *Enterococcus faecalis*, *Staphylococcus aureus*, and *Staphylococcus saprophyticus* were found in all age groups of diabetic and non-diabetic UTI patients. The findings showed that the most resistant bacteria from patients with non-diabetic UTIs were found to be resistant to amoxicillin (37.7%) and ampicillin (40%), while the most resistant bacteria from patients with diabetes were found to be resistant to tetracycline (43.3%) and cephalothin (43.5%). In patients with UTIs, ciprofloxacin was found to be the most effective antibiotic against all bacterial species.

**Discussion:**

According to the results, we concluded that the UTI etiological profiles varied among different ages. Ciprofloxacin is a safe medication with optimal sensitivity that can be used to treat both gram-positive and gram-negative bacteria.

## Introduction

Urinary tract infection (UTI) is defined as the ability of certain microorganisms to infect the tract through which urine flows to the outside after overcoming the natural immune defenses. Such an infection could occur anywhere along the urinary tract, including the bladder (cystitis), kidneys (pyelonephritis), ureters, and urethra (urethritis) (Barnett and Stephens, 1997; Detweiler et al., 2015). Urinary tract infections are currently considered among the most commonly diagnosed infectious diseases in the outpatient clinic. Hundreds of millions of people are diagnosed with UTI each year, accounting for a huge consumption of antimicrobial drugs worldwide (Ahmed et al., 2018; Sanyal et al., 2019).

Although the genitourinary tract is considered free of microorganisms, the terminal areas of the urethra are colonized with occupant microflora. From this fecal microflora of the host urethra, most UTIs originate (Bacheller and Bernstein, 1997; Wang et al., 2013). Enteric pathogenic gram-negative bacteria usually cause the majority of UTIs. *Escherichia coli* is the main causal agent of UTI (accounting for more than 80% of the infections), particularly among young females (Hirakawa et al., 2019; Klein and Hultgren, 2020). Other bacteria such as *Staphylococcus saprophyticus*, *Klebsiella pneumoniae*, *Proteus mirabilis*, and *Enterococcus faecalis* are occasionally involved (Flores-Mireles et al., 2015; Stefaniuk et al., 2016; Fernando et al., 2017).

Because of the anatomical and physiological features of the female urethra, women are more susceptible to urinary tract infections. In their lives, more than 60% of women will get a UTI, and 20–30% of them will get another one in the next six months (Huang et al., 2022). This is due to the much shorter urethra in women, positioning it very close to the anal opening, which is a constant source of fecal bacterial contamination (Kunin, 1994; Fihn, 2003). Another main aspect that raises women’s chances of contracting UTIs is diabetes. Female patients who suffer from diabetes have an increased risk of several diseases because of crucial immune inhibition (Schneeberger et al., 2014; Schneeberger et al., 2018).

Before treating UTIs, practitioners must be aware of the regionally prevalent species and their antibiotic sensitivity patterns. Unfortunately, a drastic change in the antibiotic resistance pattern of pathogenic urinary tract isolates has been observed over the past few years (Fernando et al., 2017; Milovanovic et al., 2019). Regardless of the widespread availability of antibiotics, several recent studies reported an increased resistance among UT-pathogens to antimicrobial agents commonly used to treat UTI (Costa et al., 2018; Fatima et al., 2018; Al-Naqshbandi et al., 2019; Koksal et al., 2019). Such antimicrobial resistance in UTIs is now becoming an alarming concern.

The majority of *in vitro* data on the etiology and antibiotic susceptibility profiles of UTIs in diabetic patients comes from laboratory-based surveys. Such surveys frequently focus on certain sexes, ages, clinical syndromes, or locations of the people from whom urine specimens are obtained. As a result, the stated results may differ based on many parameters related to the study sample. Therefore, the purpose of this study was to determine which bacterial species caused UTIs in female patients, both diabetic and non-diabetic, who were attending the maternity and children’s hospital in Jeddah, Saudi Arabia. Also, this work sheds some light on the distribution of antibiotic resistance within the isolated species. In addition, any significant link between the bacterial isolates in the two studied groups and antibiotic susceptibility should be noted.

## Materials and methods

### Participants and study location

Over twenty-eight months, from early January 2021 to the end of June 2023, a total of 2,245 female patients—420 of whom had diabetes—attending the inpatient and outpatient clinic of the Maternity and Children Hospital in Jeddah, Saudi Arabia, were enrolled in the study. The Ministry of Health regards this hospital as the medical authority for women’s diseases, obstetrics, gynecology, and pediatrics in Mecca Province (including its twelve governorates), which is situated in the western part of Saudi Arabia. All of the included cases in this study were newly diagnosed, had a positive culture, and displayed various signs of symptomatic UTI. None of the patients had a history of previous UTI infections or relapses, and none of the female patients included in this study had received antibiotic treatment before sample collection.

### Sample collection and examination of urine specimens

Following written consent from the patients, a single clean-catch mid-stream urine sample per patient was collected in the morning using a sterile container. Each sample was properly labeled with the relevant information and transported to the laboratory within two hours after collection. Upon receipt, urine samples were divided into three aliquots. The first aliquot was subjected to preliminary microbiological identification based on cultural characteristics on blood agar, MacConkey agar, eosin methylene blue agar, and mannitol salt agar growth media (Difco™, Detroit, MI, USA). Following a 37°C incubation period, plates were checked for bacterial growth, and a colony count was performed to determine the quantity of colony-forming units per milliliter of urine. The isolated pure colonies were subjected to Gram staining and examined under the microscope to visualize basic cell morphology. In accordance with the guidelines for differentiating between true infection and contamination, the analysis included only positive culture of a single bacterial species from the urine sample at a concentration of >10^5^ CFU/mL per patient (Akram et al., 2007).

The other two aliquots were used to prepare bacterial pellets; one was used for species identification by the MALDI-TOF MS, and the second was used for antimicrobial susceptibility testing (AST) by the VITEK 2 system (Torres-Sangiao et al., 2022).

### Bacterial species identification

Bacterial isolates were identified using the routine in-lab Microflex MALDI-TOF MS mass spectrometer system (Bruker, Germany). The analysis of mass spectra was performed by the MALDI Biotyper software version 3.1 using the reference database and default parameter settings. Identification scores of ≥2.0 were acceptable for accurate species identification, as defined by the manufacturer (Anwer et al., 2022).

### Antibiotic susceptibility testing (AST)

After bacterial species identification by MALDI-TOF MS was achieved, the pellet of the third aliquot was diluted in a saline solution to prepare a 0.5 McFarland turbidity standard for susceptibility testing to different antibiotics by the VITEK-2 system (BioMérieux, France). The tested antibiotics included the following: ciprofloxacin (CIP) (0.02–4 μg/mL), tetracycline (TET) (0.5–16 μg/mL), nalidixic acid (NAL) (2–128 μg/mL), Cefuroxime (CXM) (0.5–64 μg/mL), chloramphenicol (CHL) (4–32 μg/mL), Cephalothin (CEF) (0.5–32 μg/mL), ampicillin (AMP) (0.5–16 μg/mL), Amoxicillin (AMOX) (0.5–16 μg/mL), and gentamicin (GEN) (0.25–8 μg/mL). Finally, the AST results performed by VITEK-2 were interpreted as resistant (R) or sensitive (S) following the manufacturer’s protocols.

### Data analysis

Relative species Abundance (PI) was calculated according to the following equation (Ullah et al., 2023):


$$\mathrm{Relative Abundance}\;\left(\%\right)=Isi/\sum Nsi\;X\;100$$

Where, Isi = Total Number of individual spp.; ∑ Nsi = Total Number of species population.

S indicates the number of species, i specifies the abundance of species, I indicate to the individuals, and N is the total number of populations.

The number of resistant isolates for each antibiotic (%) was calculated according to the following equation:


$$\mathrm{Resistant isolates for each antibiotic}\;\left(\%\right)=N/N_{t}X\;100$$

Where, N = Number of resistant isolates for each antibiotic; N_t_ = Total number of isolates.

The number of resistant isolates (%) among each species was calculated according to the following equation:


$$\mathrm{Resistant}\;\mathrm{isolates}\;\mathrm{among}\;\mathrm{each}\;\mathrm{species}\;\left(\%\right)=N'/{N_{t}}_{'}X\;100$$

Where, N = Number of resistant isolates for each species; N_t_ = Total number of each species.

The distribution of each R isolates in age categories (%) was calculated according to the following equation:


$$\begin{array}{l} \mathrm{Distribution}\;\mathrm{of}\;\mathrm{each}\;R\;\mathrm{isolates}\;\\ \mathrm{in}\;\mathrm{age}\;\mathrm{categories}\;\left(\%\right)=N''/N_{t''}X\;100 \end{array}$$

Where, N = Total number of resistant isolates in each species for each age category; N_t_ = Total number of samples in each age category.

### Statistical analysis

All data analyses and visualization were done by Scientific and Research plot (SRplot) tool (Tang et al., 2023) and Toolkit for Biologists tools (TBtools v2.09) (Chen et al., 2023). We used the Wilcoxon test for non-parameter connection lines analysis between diabetic and non-diabetic UTIs.

## Results

### Population under study

**Figure 1:**
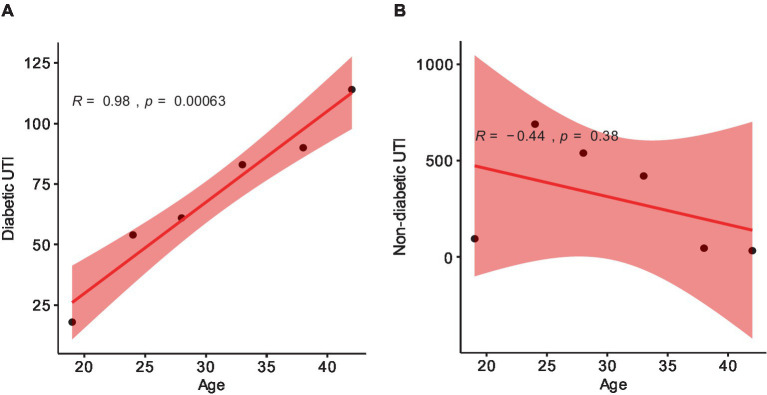
Pearson correlation coefficient (*r*) scatter plot showing the *p*-value (*p*) between: **(A)** diabetic patients and average ages; **(B)** non-diabetic patients and average ages. The range (*r*) is −1 to 1. 1 depicts totally positive correlation; −1 depicts totally negative correlation; 0 depicts no linear correlation. *p* < 0.5 is considered a non-significant value.

This study comprised 2,245 clinically diagnosed female patients of different ages with urinary tract infections (UTIs). Patients suffering from diabetes accounted for 420 (19%), whereas the rest 1825 (81%) were free of chronic conditions (Table 1). The largest incidence of UTI in non-diabetic females occurred between the ages of 22–25 (37.8%) and 26–30 (29.6%). In diabetic females, the incidence of UTI was highest among patients aged 36 to 40 (21.4%) and over 40 (27.1%) (Table 1). There is a strong positive correlation between the average age and the number of diabetic UTI patients, as demonstrated by the Pearson coefficient analysis. However, a non-significant correlation was found between the average age and the number of cases in non-diabetic female patients (Figure 1).

**Table 1:** Age distribution among UTI-positive non-diabetic and diabetic female patients.

Age Group	Non–Diabetic	Diabetic	Total
18 to 21	95	18	113
22 to 25	690	54	744
26 to 30	540	61	601
31 to 35	421	83	504
36 to 40	46	90	136
> 40	33	114	147
Total	1825	420	2,245

### Identification of UTI-causing bacterial isolates

All bacterial isolates were effectively identified using the MALDI-TOF/MS analysis after the morphological characterization of culture-positive urine samples from UTI patients. Based on the results, bacterial isolates from urine samples were divided into two groups. The first group included Gram-negative rods (84.7%), while Gram-positive cocci (15.3%) comprised the second group (Table 2).

**Table 2:** Identity of UTI encountered bacterial species isolated from the female patients.

Bacterial species		Number of isolates	Total number
Gram-negative rods	*Escherichia coli*	916	1903 (84.7%)
	*Proteus mirabilis*	231	
	*Pseudomonas aeruginosa*	197	
	*Citrobacter freundii*	149	
	*Acinetobacter baumannii*	139	
	*Klebsiella pneumonia*	119	
	*Enterobacter aerogenes*	76	
	*Enterobacter cloacae*	38	
	*Serratia marcescens*	38	
Gram-positive cocci	*Enterococcus faecalis*	124	342 (15.3%)
	*Staphylococcus aureus*	96	
	*Staphylococcus epidermidis*	66	
	*Staphylococcus saprophyticus*	56	
	Total	2,245	

### Distribution of bacterial isolates among UTI patients

**Figure 2:**
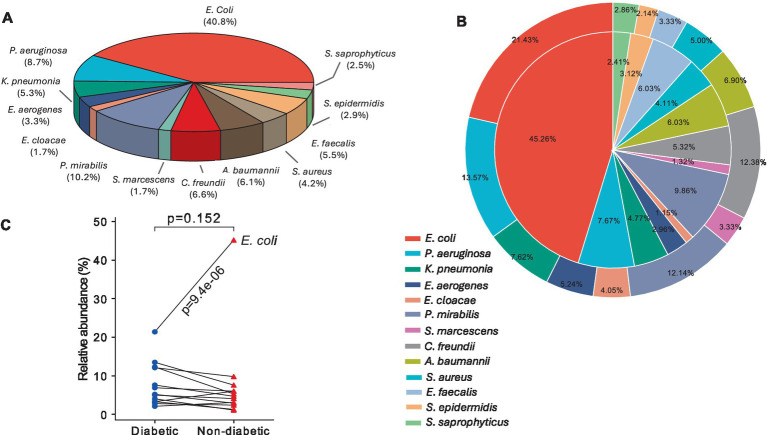
Prevalence of bacterial pathogens causing UTI among female patients: (A) Pie chart showing the total number of bacterial isolates isolated from UTI patients, (B) two-layer 2D pie plot showing the bacterial isolates among diabetic UTI patients (outer layer) and the pathogens among non-diabetic patients (inner layer), and (C) connection lines showed the relative abundance between diabetic and non-diabetic UT pathogens where p-value was calculated by Wilcoxon test (*p* < 0.5 is considered a non-significant value).

From the total number of isolates, *E. coli* (40.8%) was the predominant cause in both diabetes and non-diabetic UTI female patients, followed by *P. mirabilis*, *P. aerginosa*, and *Citrobacter freundii* (10.2, 8.7, and 6.6%, respectively). *Serratia marcescens* and *Enterobacter cloacae* had the lowest incidence (1.7%) among diabetic and non-diabetic female UTI patients (Figure 2A). *E. coli*, *E. faecalis*, and *Staphylococcus epidermidis* isolates were more common in non-diabetic (45.3, 6, and 3.1%, respectively) than in diabetic ones (21.4, 3.3, and 2.14%, respectively). The prevalence of *C. freundii* is significantly (*p* = 0.002) higher in diabetic than non-diabetic UTI patients. The remaining species were more common in diabetic female patients (Figure 2B). Figure 2C shows that there is no strong statistically significant (*p* = 0.152) difference in the distribution of isolates between diabetic and non-diabetic UTI patients. Separately, certain isolates exhibited a significant difference in prevalence between diabetic and non-diabetic UTI patients, the most obvious being *E. coli* (more prevalent in non-diabetic than diabetic UTI patients with a significant value smaller than 0.0001, Figure 2C).

### Frequency of bacterial isolates among different age groups

**Figure 3:**
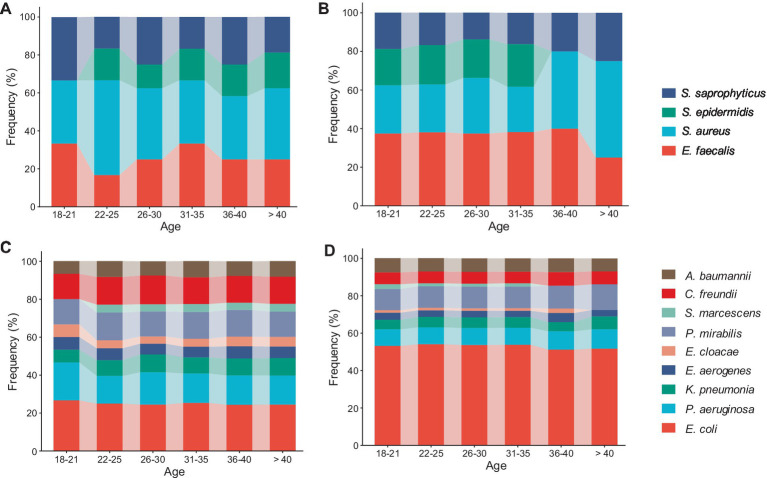
Filled stackbar charts showed the distribution of urinary pathogens across different age categories (18 - > 40): (A) Frequency of gram-positive cocci among diabetic UTI patients, (B) frequency of gram-positive cocci among non-diabetic UTI patients, (C) frequency of gram-negative rods among diabetic UTI patients, and (D) frequency of gram-negative rods among non-diabetic UTI patients.

Age-specific results revealed that all age groups of diabetic and non-diabetic UTI patients were infected with gram-positive *E. faecalis*, *S. aureus*, and *S. saprophyticus* species (Figures 3A,B). It was shown that *S. aureus* was more common in non-diabetic female patients over 40 years of age, as well as in the majority of diabetes age groups, whereas *E. faecalis* was more predominant in non-diabetic female patients between the ages of 18 and 40 (Figures 3A,B). Furthermore, no *S. epidermidis* isolates were found in diabetic female patients aged 18 to 21 years (Figure 3A) or elderly non-diabetic patients aged 36 to >40 years (Figure 3B). Among patients without diabetes of all ages, *E. coli* accounted for almost half of all gram-negative bacilli (Figure 3D). There is nearly an equal distribution of *E. coli*, *P. aeruginosa*, *C. freundii*, and *P. mirabilis* across all ages of diabetic patients (Figure 3C). *S. marcescens* and *E. cloacae* were the least common gram-negative species, and they did not span all age categories. Patients with diabetes were shown to have a more uniform distribution of their frequencies compared to those without the disease (Figures 3C,D).

### Evaluation of antibiotic resistance

To detect the existence of antibiotic-resistant bacterial pathogens among patients with UTIs (diabetic and non-diabetic), all isolates from urine samples were subjected to the VITEK-2 system. The results revealed that the highest rate of resistance among bacterial isolates from non-diabetic UTI female patients was observed against Amoxicillin (37.7%) and Ampicillin (40%). In contrast, the highest rate of resistance among diabetic UTI isolates was observed against cephalothin (43.5%) and tetracycline (43.3%). Overall, the highest antibiotic resistance was detected against amoxicillin (38.2%) and ampicillin (39.5%) in non-diabetic and diabetic bacterial isolates (Table 3). Ciprofloxacin and nalidixic acid were the most effective antibiotics against bacterial isolates in diabetic and non-diabetics (Table 3).

**Table 3:** Susceptibility profile of bacterial isolates from diabetic and non-diabetic UTI against different antibiotics.

Antibiotic	Non-Diabetic 1825		Diabetic 420		Total 2,245
	Sensitive	Resistant	Sensitive	Resistant	Resistant
Chloramphenicol	1,462	363 (19.9%)	261	159 (37.9%)	522 (23.3%)
Nalidixic acid	1,533	292 (16%)	306	114 (27.2%)	406 (18%)
Ciprofloxacin	1,652	173 (9.5%)	320	100 (23.8%)	273 (12.2%)
Tetracycline	1,213	612 (33.5%)	238	182 (43.3%)	794 (35.4%)
Cefuroxime	1,396	429 (23.5%)	282	138 (32.9%)	567 (25.3%)
Cephalothin	1,286	539 (29.5%)	237	183 (43.5%)	722 (32.2%)
Amoxicillin	1,137	688 (37.7%)	251	169 (40.5%)	857 (38.2%)
Ampicillin	1,096	729 (40%)	264	156 (37.1%)	885 (39.5%)
Gentamicin	1,228	597 (32.7%)	275	145 (34.5%)	742 (33%)

**Figure 4:**
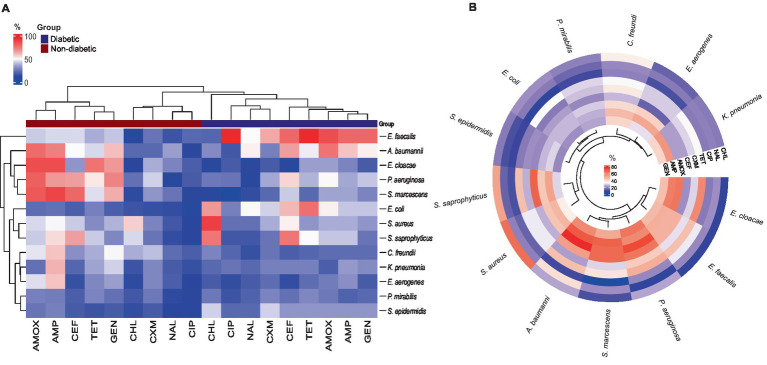
Antibiotic-resistant profile of bacterial pathogens: (A) Heatmap illustrating the percentage of antibiotic resistance between diabetic and non-diabetic patients, with hierarchical clustering applied to both rows and columns. (B) Circular heatmap showing the antibiotic resistance for all isolates among the UTI patients, also featuring clustering to highlight patterns in resistance profiles.

The *E. faecalis* isolates in diabetes patients showed the highest resistance rate to all antibiotics, except for chloramphenicol. It was also discovered that every *E. faecalis* isolate was resistant to ciprofloxacin and tetracycline (Figure 4A). The heatmap clustering revealed that in non-diabetic individuals, isolates of *P. aeruginosa*, *Acinetobacter baumannii*, *E. cloacae*, and *S. marcescens* had a significant profile of antibiotic resistance against amoxicillin, ampicillin, and gentamicin (Figure 4A). Particularly in diabetic patients, chloramphenicol showed exceptional activity against all isolates except for *E. coli*, *S. aureus*, and *S. saprophyticus*. The isolates of *P. mirabilis* and *S. epidermidis* showed the lowest rates of resistance to the antibiotics under study (Figure 4A). An examination of the average rate of resistance among the remaining bacterial UTI pathogens revealed that *C. freundii*, *K. pneumonia*, and *Enterobacter aerogenes* showed modest degrees of resistance toward ampicillin in non-diabetic persons (Figure 4A).

The most effective antibiotic against all bacterial isolates in UTI patients was found to be ciprofloxacin. Nalidixic acid and cefuroxime also demonstrated high levels of effectiveness against the isolates, with the exception of *A. baumannii* in the case of nalidixic acid and *P. aeruginosa* and *C. freundii* in the case of cefuroxime (Figure 4B). The remaining five antibiotics—tetracycline, amoxicillin, ampicillin, cephalothin, and gentamycin—exhibited different antibiogram patterns in UTI patients when compared to bacterial isolates, with a higher rate of resistance (lower efficacy) demonstrated (Figure 4B). Additionally, Figure 4B results showed that the cluster of bacteria that included *S. epidermidis*, *P. mirabilis*, and *E. coli* was more sensitive to every tested antibiotic. Lastly, *C. freundii*, *P. aeruginosa*, and *K. pneumonia* showed moderate resistance against amoxicillin, ampicillin, and gentamycin.

**Figure 5:**
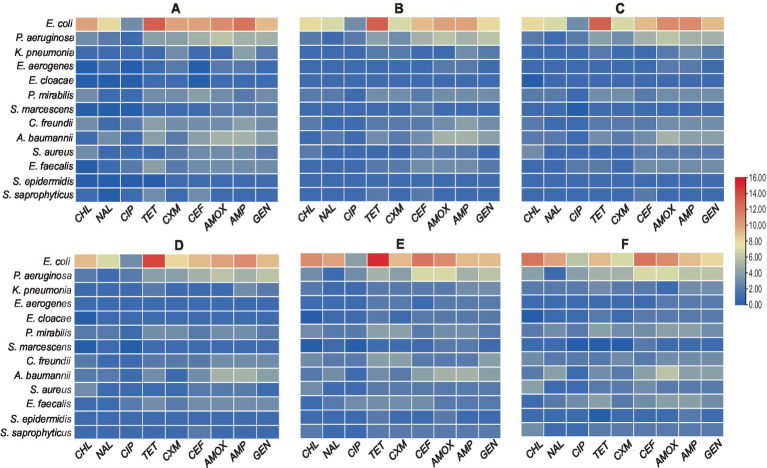
Heatmaps showing antibiogram profiles revealing a relationship between antibiotic-resistant isolates and age categories: (A) resistant bacterial isolates among ages from 18 to 21 years, (B) resistant bacterial isolates among ages from 22–25 years, (C) resistant bacterial isolates among ages from 26–30 years, (D) resistant bacterial isolates among ages from 31–35 years, (E) resistant bacterial isolates among ages from 36–40 years, and (F) resistant bacterial isolates among ages from >40 years.

We created an antibiogram matrix that provided preliminary data on the relationship between the number of resistant bacterial isolates and the total number of samples within each age category. The aim of Figure 5 was to provide a quick data analysis indicator of the resistance profile for each age group. This was accomplished by calculating the ratio of resistant isolates to total isolates within each age category, allowing for an evaluation of differences among the six groups. Notably, *E. coli* exhibited decreased resistance to chloramphenicol, nalidixic acid, and tetracycline. Despite these exceptions, the color coding of all six antibiograms showed significant symmetry. Although the results did not reveal any substantial differences in the antibiogram patterns, they still provided valuable feedback, indicating a similar resistance profile across the age range (18–40).

## Discussion

Despite the fact that most urinary tract bacterial infections are acute and temporary and that antibiotics are widely available, they remain the most common and cause a significant amount of morbidity in the community. The etiology and antibiotic susceptibility patterns have evolved over time and place and will persist in doing so (Chu and Lowder, 2018; Ahmed et al., 2019; Anwer et al., 2022).

This study was conducted to evaluate the distribution of UTI in females and to review the antibiotics that can be used for the treatment. A total of 2,245 urine samples from female UTI patients of various ages were obtained throughout the course of the two-and-a-half-year study period, with 420 (19%) of them being diabetic. The isolated gram-positive and gram-negative pathogenic bacteria were identified by MALDI-TOF/MS, the most accurate, rapid, cost-effective, and affordable technique in clinical laboratories for bacterial identification (Zhou et al., 2014; Yogo et al., 2022). Consistent with previous studies conducted in this area, the findings indicated that both gram-positive and gram-negative bacterial species were implicated in causing urinary tract infections with different percentages in both diabetic and non-diabetic patients (Al-Asoufi et al., 2017; Sain et al., 2022).

According to both Aswani et al. (2014) and Bonadio et al. (2006), there was no significant correlation between age and the incidence of UTI in diabetic and non-diabetic patients. Nevertheless, in this study, female patients with diabetes showed a strong positive correlation between the average age and the number of diabetic UTI patients. Patients over 40 (27.1%) and those between the ages of 36 and 40 (21.4%) had the highest prevalence of UTI, most likely as a result of their severely weakened immune systems brought on by diabetes. Still, similar to the observations made by the previously mentioned authors, our study also reported a non-significant correlation between the average age and the number of cases in non-diabetic female patients. Such insignificance can be caused by factors like sexual activity and delayed postcoital voiding, which can introduce bacteria into the vaginal orifice, where they stay and multiply in the bladder. Other possible causes include using a diaphragm birth control method, experiencing cystitis, which affects 27% of healthy women, and certain antimicrobial agents, which can alter the vaginal microflora and make it easier for urinary tract pathogens to survive (Storme et al., 2019).

The study showed that *E. coli* (40.8%) was the most common bacteria isolated from urine samples in our investigation. Other studies have also reported a similar frequency of UTI caused by *E. coli* (Al-Asoufi et al., 2017; Kumar et al., 2019; Ramrakhia et al., 2020). Although *E. coli* in this study was still the dominant urinary pathogen, the percentage was lower than the report from mainland China (50%) (Qiao et al., 2013), Taiwan (60.5%) (DeRosa et al., 2021), and Ethiopia (52.7%) (Bitew et al., 2017). The reason *E. coli* has a higher incidence than other UTI-causing pathogens could be that *E. coli* is present in large quantities, increasing their chances of being implicated in the illness. Furthermore, *E. coli* has many virulence factors that increase the likelihood of *E. coli* adhering to the urothelial tissue (the most essential step in generating UTI) and inducing clinical inflammation. While *E. coli*-associated UTIs were the most common in both groups, the non-diabetic group’s total incidence of *E. coli*-associated UTIs was much greater (45.3%) than that of the diabetic group (21.4%). The recent findings by Ho et al. (2021) showing insulin treatment in patients with diabetes plays a vital role in downregulating *E. coli*-caused UTI infections could be the explanation of this inconsistency in the results. Apart from *E. coil*, all the remaining bacterial species exhibited a variable percentage among the diabetic UTI patients with non-noticeable significance.

The findings of our study also showed that *Proteus mirabilis* and *Pseudomonas aeruginosa* ranked second and third, respectively, behind *E. coli* as the causative agents of UTI with rates of 8.7 and 10.2%, whereas *K. pneumoniae* was only responsible for 5.3% of all UTI cases. However, prior research has shown that among the gram-negative bacterial agents linked to urinary tract infections, *E. coli* is typically most common, followed by *K. pneumoniae*, *P. mirabilis*, and *P. aeruginosa* in that order (Flores-Mireles et al., 2015; Huang et al., 2022; Islam et al., 2022; Hamadalneel et al., 2024). In the same context, our findings ran counter to the reports of several authors (Gessese et al., 2017; Chelkeba et al., 2022) who claimed that gram-positive *S. aureus* and *S. saprophyticus* bacterial species were primarily responsible for UTIs in women. This is because the *S. saprophyticus* bacterial isolates in this study were much less likely to cause UTI in either group. However, our study’s findings were in line with those of AlShamlan et al. (2022), who carried out a comparable survey on pregnant women in Saudi Arabia and found that *E. faecalis* was the most isolated bacteria from urine samples. These parallels and discrepancies in the variety and distribution of bacteria that cause urinary tract infections (UTIs) can be attributed to various environmental circumstances, host characteristics, socioeconomic norms, and cleanliness habits within each society. In agreement with several other studies that represented similar results indicating that *E. aerogenes, E. cloacae, S. marcescens,* and *S. epidermidis* percentages of isolation from UTI cases are generally low (Mohammed et al., 2016; Yogo et al., 2022; Alhhazmi et al., 2024).

The distribution of bacterial pathogens responsible for UTIs in relation to age was also clarified by the current study. According to the study, *S. aureus* was more common in female patients over 40 years old, while gram-positive bacteria, particularly *E. faecalis*, showed a noticeable increase in prevalence among younger females. These results were in opposition to those of previous studies (Niu et al., 2023; Zhan et al., 2024) which showed that the most prevalent bacteria in these age groups were *Enterococcus faecalis* and *Enterococcus faecium*. Regardless, the most frequent pathogens responsible for UTIs stay the same, though the order fluctuates a little bit between regions. As more than half of all gram-negative bacilli in all age groups with UTIs, *E. coli* was shown to have a high prevalence in the study’s age-related relationships. Our knowledge of UTIs is further complicated by these age-related patterns of bacterial infections. Age-specific management options are critical for improving healthcare outcomes, as evidenced by the significant changes in UTI bacterial prevalence with age (Magliano et al., 2012).

The diversified etiological profile of gram-negative species, such as *S. marcescens* and *E. cloacae*, was also noticeable. This was due to the observation that the frequencies of these species were more equally distributed in individuals with diabetes, but the diversity of these species was low and not detected across all age groups in non-diabetics. The absence of *S. epidermidis* isolates was observed in female diabetic patients aged 18 to 21 years as well as in elderly non-diabetic patients aged 36 to >40 years; this was probably because the number of isolates was the smallest among all age categories. Lastly, there was no discernible variation in the incidence of bacterial infections associated with UTIs among age groups.

UTIs vary in location and alter over time due to the dissemination of antibiotic-resistant bacteria. Such resistance is typically contingent upon the colonizing organisms and the antimicrobial usage pattern. Over the past few decades, the spectrum of pathogenic bacteria isolated from patient urine around the world has remained largely unchanged, but most countries have seen dramatic changes in the sensitivity profile and resistance pattern (Majumder et al., 2022; Mancuso et al., 2023; Sula et al., 2023). Antibiotic resistance is a prevalent occurrence in underdeveloped nations when medications are easily accessible without a prescription. Each nation has a different resistance pattern (Khilnani et al., 2024). To determine which antibiotic would be best for treating individuals with a urinary tract infection in its early stages, all isolated gram-negative and positive bacterial species were exposed to a range of antibiotics. To find out the isolates’ sensitivity profile, the VITEK-2 system used a variety of antibiotics from different families. Similar to previous studies, the sensitivity and resistance level toward the commonly used antibiotic differs depending on the bacterial species and the mechanism of action of these antibiotics (Plate et al., 2019; Khan et al., 2023).

Ciprofloxacin and nalidixic acid were the most efficient medications against the isolated bacterial species from UTI patients with and without diabetes. The two antibiotics are members of the same family (synthetic quinolone-type antibiotics), and they work similarly in inhibiting DNA replication by interfering with the enzymes that cause DNA to supercoil (Hiasa, 2018). In a similar study conducted in Saudi Arabia on female patients with UTI, ciprofloxacin antibiotic was deemed the most suitable treatment choice (Darraj, 2023). The strong clinical value of ciprofloxacin, a second-generation fluoroquinolone, arises from the fact that it does not exhibit cross-resistance with other antibiotics (Pietsch et al., 2017; Zhang et al., 2018). However, because of growing bacterial resistance, nalidixic acid—the first quinolone to be identified—has been stopped as a therapy for urinary tract infections (Tang and Zhao, 2023). Nevertheless, the current study’s findings showed that nalidixic acid had a high activity level against gram-negative and gram-positive UTI isolates. The resurgence of nalidixic acid parent molecules has been studied in several recent research, and these findings corroborate such efforts (Dwivedi et al., 2015; Pandey et al., 2018; Gaurav et al., 2021).

Concurring with the results of Firissa et al. (2023), amoxicillin and ampicillin (aminopenicillin family) exhibited the highest level of antibiotic resistance among the isolated species in both non-diabetic and diabetic individuals. This is most likely because these isolates can manufacture the *β*-lactamase enzyme, which targets the β-lactam ring in these antibiotics’ structures and gives them their activity. Therefore, these antibiotics should be used in conjunction with a β-lactamase inhibitor, a resistance buster, for treating UTIs (Rutten et al., 2020). The empirical use of a new generation of β-lactam antibiotics (cephalosporins), is a significant contributing factor to antibiotic resistance and increases the chance of generating multidrug-resistant bacteria (Gashe et al., 2018). Cephalothin and cefuroxime, both popular medications for UTIs, serve as excellent examples. Nevertheless, in this study, the use of both antibiotics has revealed multiple resistance patterns among uropathogenic isolates. Such a high rate of resistance indicated that it should be reevaluated as a first-line treatment.

Previous studies have shown that UTI-causing bacteria are typically susceptible to antibiotic classes that block protein synthesis, including gentamicin and tetracycline. Unfortunately, an increased rate of resistance to both antibiotics was found in this study. This could be the result of either the inconsistent and improper use of those medications over an extended period of time, or it could be the result of strain-level variation within the isolated species, the hospitals’ geographic location, the source of the infection, and patient factors, which might all explain the variation in susceptibility data results (Cunha et al., 2018; Vazouras et al., 2018).

The isolates of *P. aeruginosa* and *A. baumannii* exhibited the highest rate of resistance. These findings are consistent with earlier research that suggests plasmids, which are rapidly transferred between isolates and carry resistance genes to numerous antibiotics, may be responsible for the isolates’ high resistance capacity (Bagińska et al., 2021; Bhargava et al., 2022). Furthermore, it was discovered that, of all the gram-positive bacterial isolates, *E. faecalis* was the most resistant to all antibiotics with the exception of chloramphenicol. According to Sharon et al. (2023), distinct chromosomal changes and a number of well-known genes that give *E. faecalis* species intrinsic resistance to antibiotics were the causes of the over ten drug classes for which *E. faecalis* resistance was predicted.

An intriguing finding in this investigation was the antibiogram of urine-isolated pathogens, which demonstrated that while *E. coli* was the most frequently isolated pathogen, every isolate had modest resistance rates to most of the medications utilized. Furthermore, the *P. mirabilis* isolates exhibited the lowest resistance rate, whereas *C. freundii, K. pneumonia*, and *E. aerogenes* showed moderate resistance. Our results are inconsistent with the results of a previous review study conducted by Zhanel et al. (2022), which showed a considerable level of resistance across all previously reported uropathogenic species. Nonetheless, it’s important to keep in mind that susceptibility studies have limitations because most UTIs are treated empirically and most medical professionals do not take a urine sample before treating an infection (de Cueto et al., 2017). As a result, it is more likely that the urine samples used in various research came from individuals who had previously received antibiotic treatment for a UTI that was either recurrent or prior. However, none of the patients in our study had a history of prior UTI infections or relapses, which may account for the high degree of antibiotic sensitivity.

Finally, the antibiogram matrix developed for this investigation showed no noticeable correlations between the total number of resistant isolates within each age group and the number of resistant bacterial isolates. This result aligns with a study by Alrebish et al. (2022), which found that, in the elderly age group (≥64 years old), the number of antibiotic-resistant gram-positive and negative bacterial isolates was significantly higher than in younger patients. However, similar to our findings, there was no significant difference in number among the remaining age groups (youth and middle-aged groups). Due to their longer hospital stays than younger people, elderly people are more likely to be exposed to drug-resistant bacteria and are expected to be colonized by resistant bacterial species than young people with chronic illnesses like diabetes (Denkinger et al., 2013; Hossain et al., 2020).

## Conclusion

The ongoing investigation of the spread of uropathogens and their patterns of antibiotic susceptibility aids in tracking the development of UTIs. From our study, both gram-positive and negative bacterial species were associated with urinary tract infections in diabetic and non-diabetic patients. The study found that there are notable variations in the prevalence of UTI bacteria with age, which supports the need for age-specific therapy strategies to enhance UTI healthcare outcomes. *E. coli* is still the most frequent pathogen responsible for UTI among both diabetics and non-diabetics where it is higher significantly in non-diabetic than diabetic patients. This study has revealed the alarming level of resistance to B-lactams antibiotics against UT pathogens, while most of the isolates were sensitive to ciprofloxacin and nalidixic acid. The two bacteria with the highest rates of resistance to the majority of the tested antibiotics were *P. aeruginosa* and *A. baumannii.* There were no discernible relationships found between the total number of resistant isolates in each age group and the number of resistant bacterial isolates. This is one of the few studies comparing female patients with UTIs of various ages who are diabetic and non-diabetic, which will serve as proof and better support future research.

## Data Availability

The original contributions presented in the study are included in the article, further inquiries can be directed to the corresponding author.
